# Supplementary material for: Optimized AAV Vectors for TMC1 Gene Therapy in a Humanized Mouse Model of DFNB7/11
Source: Biomolecules. 2022 Jun 29;12(7):914. doi: 10.3390/biom12070914 (PMC9313133; doi:10.3390/biom12070914)
Supplement: Supplementary file 1 [file biomolecules-12-00914-s001.zip › SM.pdf]

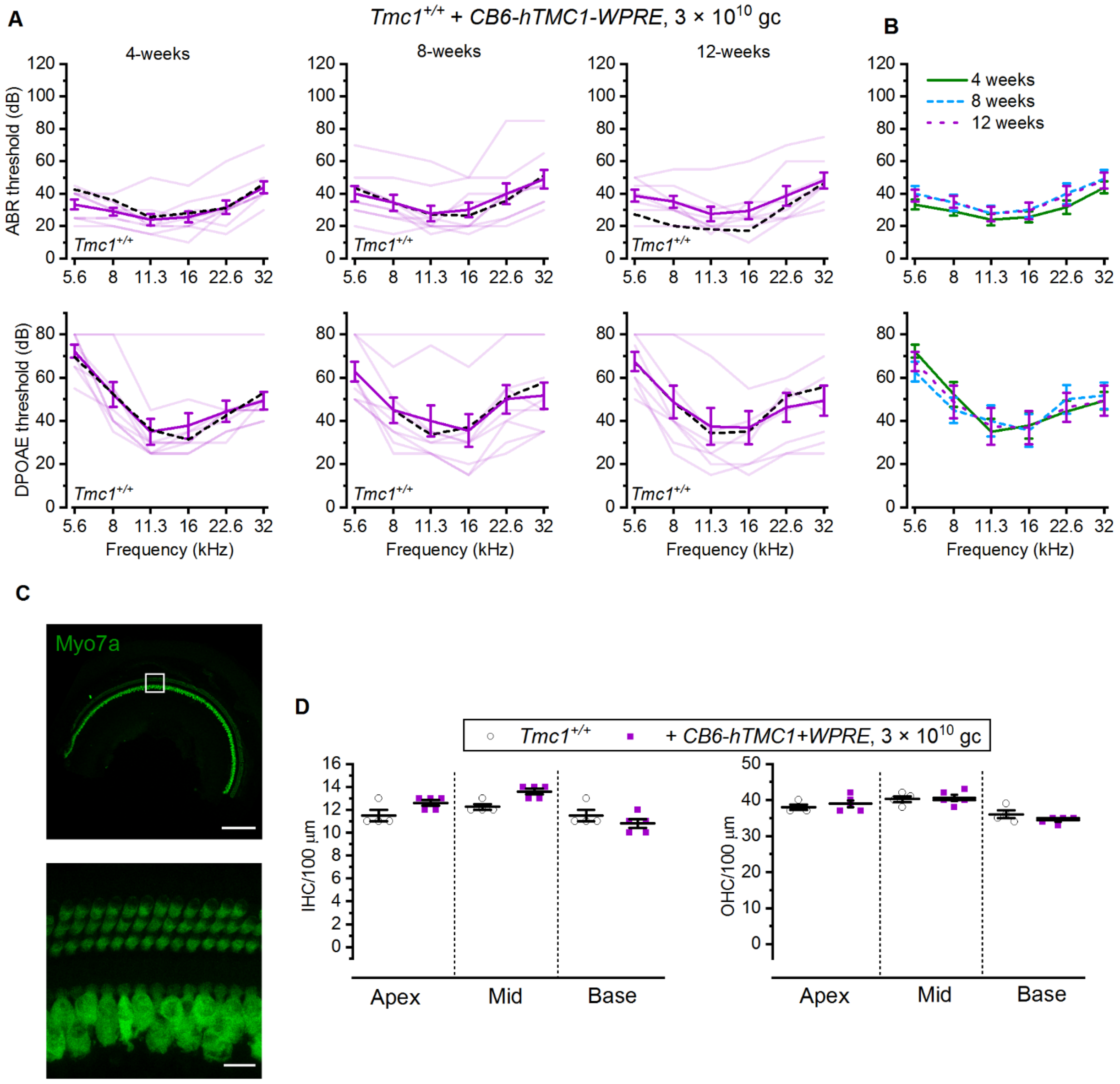

Figure S1. Wild-type mice injected with AAV2/9-PHP.B-*CB6-hTMC1+WPRE* have normal hearing. (**A**) ABRs (above) and DPOAEs (below) as a function of frequency of *Tmc1*$^{+/+}$ mice injected with the *CB6-hTMC1+WPRE* construct (3 × 10^{10} gc) at 4 (left), 8 (center) and 12 weeks (right) of age. Mean ± S.E.M. thresholds are shown in bold traces and individual recordings are shown as lighter traces. Dashed lines indicate mean ABR or DPOAE thresholds for uninjected *Tmc1*$^{+/+}$ mice. (**B**) Mean ± S.E.M. ABR (above) or DPOAE (below) thresholds at each individual timepoint are shown in a single plot. (**C**) Representative confocal images from middlecochlear sections of *Tmc1*$^{+/+}$ mice injected with *CMV-hTMC1* (3 × 10^{10} gc) from 12-week-old mice immunostained against myosin 7a. Scale bars: upper panels (10×) 200 µm; lower panels (63×) 15 µm. (**D**) Mean ± S.E.M hair cell counts per 100 µm sections for IHCs (left) and OHCs (right) from untreated (white circles) and injected *Tmc1*$^{+/+}$ (purple squares) mice. Individual samples are included in the scatterplots.

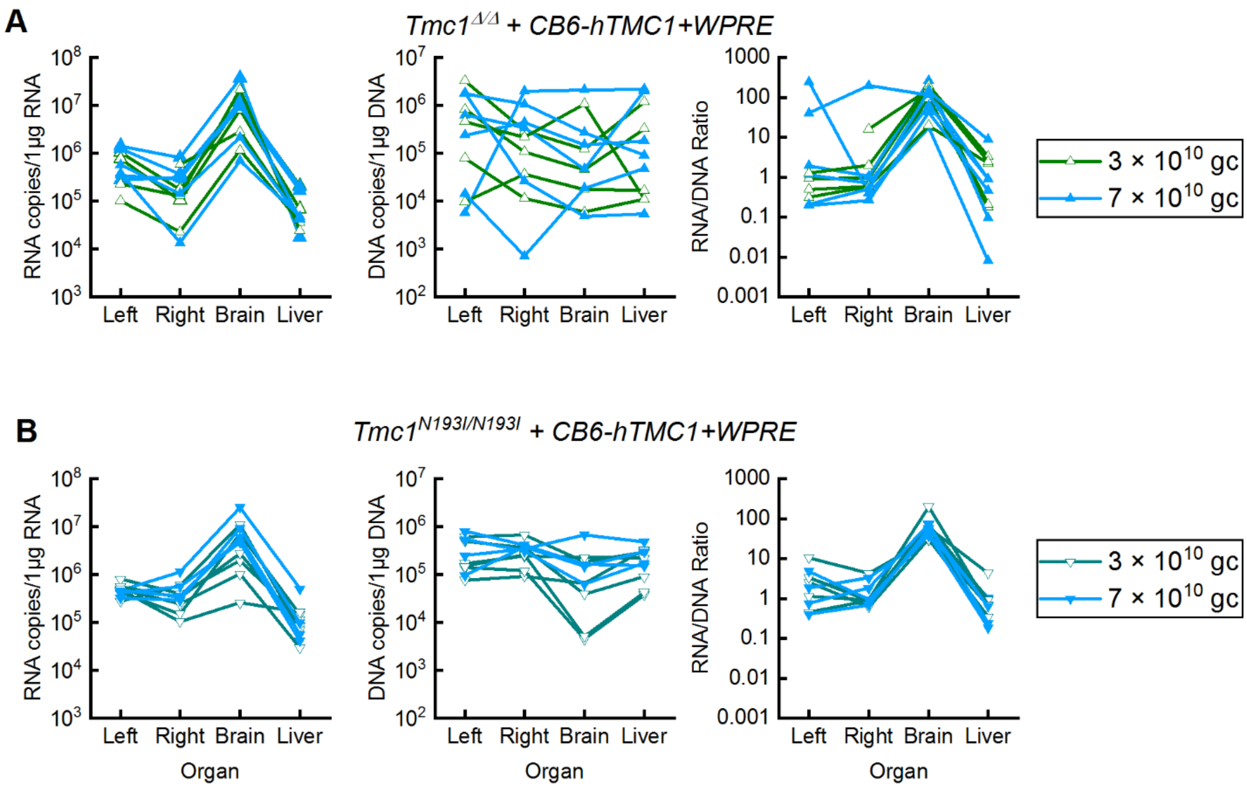

Figure S2. Viral nucleic acid biodistribution profile in target and off-target mouse tissue. Viral RNA (left) or DNA (center) concentration (copies per 1 µg of DNA/RNA) in (**A**) *Tmc1*$^{Δ/Δ}$ or (**B**) *Tmc1*$^{N193I/N193I}$ mice injected with AAV9-PHP.B-*CB6-hTMC1+WPRE* in the left (injected) or right (contralateral) inner ears, the brain and liver. RNA to DNA ratio (RNA/DNA) is shown (right). Mice injected with the 3 × 10^{10} gc dose are shown using green triangles and those injected with 7 × 10^{10} gc dose are shown using light blue filled triangles. Measurements from the same animal are linked by connecting lines.

Tables S1-S6. Values and statistics for data presented in Figures 1-5 and Supp. Figure S1, respectively.
